# Switching to Dupilumab from Other Biologics without a Treatment Interval in Patients with Severe Asthma: A Multi-Center Retrospective Study

**DOI:** 10.3390/jcm12165174

**Published:** 2023-08-09

**Authors:** Hisao Higo, Hirohisa Ichikawa, Yukako Arakawa, Yoshihiro Mori, Junko Itano, Akihiko Taniguchi, Satoru Senoo, Goro Kimura, Yasushi Tanimoto, Kohei Miyake, Tomoya Katsuta, Mikio Kataoka, Yoshinobu Maeda, Katsuyuki Kiura, Nobuaki Miyahara, Okayama Respiratory Disease Study Group (ORDSG)

**Affiliations:** 1 Department of Allergy and Respiratory Medicine, Okayama University Hospital, Okayama 700-8558, Japan; prea4jsb@s.okayama-u.ac.jp (H.H.);; 2 Department of Hematology, Oncology, and Respiratory Medicine, Okayama University Graduate School of Medicine, Dentistry, and Pharmaceutical Sciences, Okayama 700-8558, Japan; 3 Department of Internal Medicine, Kagawa Rosai Hospital, Marugame 763-8502, Japan; 4 Department of Respiratory Medicine, KKR Takamatsu Hospital, Takamatsu 760-0018, Japan; 5 Department of Allergy and Respiratory Medicine, National Hospital Organization Minami-Okayama Medical Center, Okayama 701-0304, Japan; 6 Department of Respiratory Medicine, National Hospital Organization Himeji Medical Center, Himeji 670-8520, Japan; 7 Department of Respiratory Medicine, Ehime Prefectural Central Hospital, Matsuyama 790-0024, Japan; 8 Department of Respiratory Medicine, Onomichi Municipal Hospital, Onomichi 722-8503, Japan; 9 Department of Medical Technology, Okayama University Graduate School of Health Sciences, Okayama 700-8558, Japan

**Keywords:** dupilumab, severe asthma, treatment interval, eosinophilic chronic rhinosinusitis

## Abstract

Background: Dupilumab is a fully humanized monoclonal antibody that blocks interleukin-4 and interleukin-13 signals. Several large clinical trials have demonstrated the efficacy of dupilumab in patients with severe asthma. However, few studies have examined a switch to dupilumab from other biologics. Methods: This retrospective, multi-center observational study was conducted by the Okayama Respiratory Disease Study Group. Consecutive patients with severe asthma who were switched to dupilumab from other biologics without a treatment interval between May 2019 and September 2021 were enrolled. Patients with a treatment interval of more than twice the standard dosing interval for the previous biologic prior to dupilumab administration were excluded. Results: The median patient age of the 27 patients enrolled in this study was 57 years (IQR, 45–68 years). Eosinophilic chronic rhinosinusitis (ECRS)/chronic rhinosinusitis with nasal polyp (CRSwNP) was confirmed in 23 patients. Previous biologics consisted of omalizumab (n = 3), mepolizumab (n = 3), and benralizumab (n = 21). Dupilumab significantly improved FEV_1_ (median improvement: +145 mL) and the asthma control test score (median improvement: +2). The overall response rate in patients receiving dupilumab for asthma as determined using the Global Evaluations of Treatment Effectiveness (GETE) was 77.8%. There were no significant differences in the baseline characteristics of the GETE-improved group vs. the non-GETE-improved group. ECRS/CRSwNP improved in 20 of the 23 patients (87.0%). Overall, 8 of the 27 patients (29.6%) developed transient hypereosinophilia (>1500/μL), but all were asymptomatic and able to continue dupilumab therapy. Conclusions: Dupilumab was highly effective for the treatment of severe asthma and ECRS/CRSwNP, even in patients switched from other biologics without a treatment interval.

## 1. Introduction

Asthma is a common disease that affects 300 million people worldwide [1]. The estimated prevalence of severe asthma is 3–10% of the total asthmatic population [2]. As the molecular mechanisms involved in the pathogenesis of asthma have been gradually elucidated, new biological therapies have been developed to treat severe asthma. The first biologic for severe asthma was the anti-IgE monoclonal antibody omalizumab, which suppresses allergic reactions by inhibiting IgE binding to high-affinity receptors on mast cells and basophils [3]. Omalizumab has been shown to reduce the rate of asthma exacerbation significantly [4]. The anti-interleukin-5 (IL-5) monoclonal antibody mepolizumab and the anti-IL-5 receptor α (IL-5Rα) monoclonal antibody benralizumab, both available in Japan, deplete eosinophils by binding to IL-5 or IL-5Rα, thus ameliorating eosinophilic inflammation of the airways in asthma patients [5].

Dupilumab (Dupixent^®^) is the fourth biologic to become available in Japan since 2018. It is a fully humanized monoclonal antibody of the IgG4 type, and it recognizes the alpha subunit of the IL-4 receptor α (IL-4Rα), blocking both IL-4 and IL-13 signals [6]. IL-4 induces the differentiation of type-2 helper T (Th2) cells and group 2 innate lymphoid cells (ILC2), and IgE production from B cells. Th2 cells and ILC2 produce IL-5 and activate eosinophils. IL-4 and IL-13 enhance the expression of adhesion molecules on endothelial cells and induce the production of chemokines such as eotaxin by airway epithelial cells, thereby recruiting eosinophils from the blood circulation to the airway mucosa. As a result of these mechanisms, IL-4 and IL-13 induce type-2 inflammation [7]. Several large clinical trials in patients with severe asthma have shown that dupilumab reduces asthma exacerbations, improves scores in obstructive pulmonary impairment and asthma control tests, and decreases the oral corticosteroid dose [8,9,10,11]. As the fourth biologic for asthma approved in Japan, dupilumab is likely to be administered to patients previously treated with other biologics. However, few studies have examined the effect of a switch to dupilumab from those biologics [12,13,14], and no study has focused on patients who were switched without a treatment interval. A long interval before a switch to dupilumab complicates comparisons of efficacy vs. previous biologics. Therefore, we conducted a retrospective study to examine the efficacy of dupilumab in patients who were switched from other biologics without a treatment interval.

## 2. Materials and Methods

### 2.1. Study Design

This retrospective, multi-center observational study was conducted by the Okayama Respiratory Disease Study Group. Participating institutions included Okayama University Hospital, KKR Takamatsu Hospital, National Hospital Organization Minami-Okayama Medical Center, National Hospital Organization Himeji Medical Center, Ehime Prefectural Central Hospital, Kagawa Rosai Hospital, and Onomichi Municipal Hospital. The study was approved by the institutional review boards of Okayama University Hospital (no. 2112–034) and all other participating hospitals. The requirement for written informed consent was waived because of the retrospective nature of the study. Consecutive patients with severe asthma who were switched to dupilumab at a dose of 300 mg (loading dose, 600 mg) every 2 weeks from other biologics between May 2019 and September 2021 were enrolled. Asthma was diagnosed based on the Japanese guidelines and the Global Initiative of Asthma guidelines [15,16]. ECRS/CRSwNP was diagnosed using the Japanese Epidemiological Survey of Refractory Eosinophilic Chronic Rhinosinusitis scoring system [17]. To compare the effects of previous biologics and dupilumab, patients with a treatment interval of more than twice the standard dosing interval for the previous biologic prior to dupilumab administration were excluded. Patients treated with the respective biologic for <3 months were also excluded. A high dose of inhaled corticosteroid (ICS) was defined as a total daily dose of fluticasone propionate of 1000 μg or equivalent. A medium dose of ICS was defined as a total daily dose of fluticasone propionate of 500 μg or equivalent. The clinical data of patients, including disease duration, body mass index, smoking history, allergies, comorbidities, treatment, blood eosinophil count, exhaled nitric oxide (FeNO), serum IgE level, and pulmonary function test, were collected from their medical records. Because biologics affect the levels of several biomarkers (blood eosinophil count, FeNO, and serum IgE), data prior to their administration were used to evaluate the characteristics of the study patients.

### 2.2. Study Assessments

The FeNO level was measured using a NIOX VERO™ device (Aerocrine AB, Stockholm, Sweden). The primary efficacy outcomes included the physician-reported Global Evaluation of Treatment Effectiveness (GETE) [4,18,19], in which the outcome is ranked as excellent (complete control of asthma), good (marked improvement), moderate (discernible but limited improvement), poor (no appreciable change), or worse. A GETE responder is defined as a patient with an excellent/good response to treatment with biologic agents. The GETE score determined after the introduction of the previous biologic was used if the overall evaluation did not change after the switch to dupilumab. Asthmatic symptoms were evaluated using the asthma control test (ACT), a clinically useful scoring system. This test contains five questions related to the frequency of asthma symptoms and rescue medication use during the previous 4 weeks [20]. Patients with ACT scores of 20–25 are considered to have well-controlled asthma, with a minimal clinically important difference in the ACT score of 3 points [21]. Dupilumab efficacy was evaluated between 3 months and 1 year in most cases.

### 2.3. Statistical Analysis

Statistical analyses were performed using EZR software version 1.36 (Saitama Medical Center, Jichi Medical University, Saitama, Japan) [22]. Patient characteristics were compared using Fisher’s exact test for binary variables. Continuous variables were evaluated using the Mann–Whitney U test or the Wilcoxon signed-rank test. A *p*-value < 0.05 was considered to indicate statistical significance.

## 3. Results

### 3.1. Clinical Characteristics

Table 1 shows the clinical characteristics of the 27 patients with severe asthma who were enrolled in this study. Comorbidities included allergic bronchopulmonary aspergillosis (n = 3), eosinophilic chronic rhinosinusitis (ECRS)/chronic rhinosinusitis with nasal polyp (CRSwNP) (n = 23), and eosinophilic otitis media (EOM) (n = 11). All patients were treated with ICS, and nine patients (33.3%) received maintenance OCS, including five for the treatment of asthma. Previous biologics treatments consisted of omalizumab (n = 3), mepolizumab (n = 3), and benralizumab (n = 21). The median period of treatment with these previous biologics was 421 days (interquartile range (IQR), 301–673 days). Dupilumab was administered as a second-line biologic to 19 patients, as a third-line biologic to 7 patients, and as a fourth-line biologic to 1 patient.

Levels of three biomarkers of type-2 inflammation (blood eosinophil count, serum IgE, and FeNO) before the use of any biologics are shown in Table 1. Although analysis was not performed for all cases due to missing data, the percentages of patients with a blood eosinophil count >150/μL, a serum IgE level of >167 IU/mL, and an FeNO value of >25 ppb were 80.8% (21 of 26 patients), 80.0% (16 of 20 patients), and 85.7% (18 of 21 patients), respectively. In 6 of the 27 patients, the blood eosinophil count was >1500/μL. All but one case, in which the values of all biomarkers were not obtained, were positive for at least one biomarker. Despite the previous treatment with biologics, 18 of 19 patients had FeNO values greater than 25 ppb (94.7%).

The reasons for switching biologics included persistent asthmatic symptoms, persistent ECRS/CRSwNP symptoms, persistent EOM symptoms, and self-administration (Table 2). Asthma symptoms were among the reasons for switching in only 44% of cases, while ECRS was the reason for switching in 67% of cases.

### 3.2. Efficacy of Dupilumab

**Figure 1:**
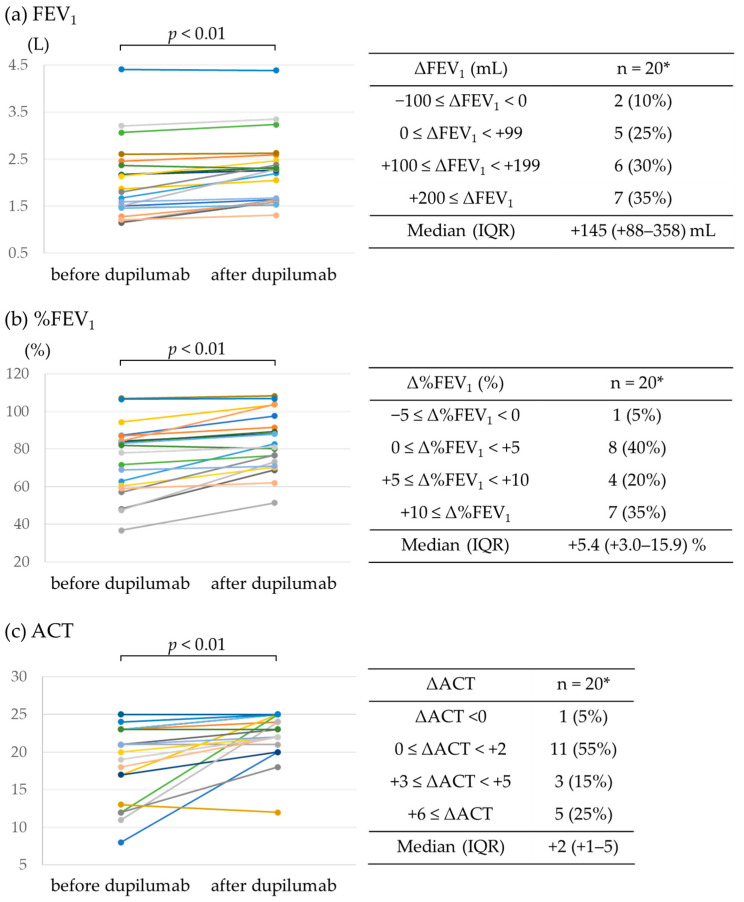
Effect of dupilumab on FEV_1_ and ACT score. (**a**) FEV_1_, (**b**) %FEV_1_, and (**c**) ACT score before and after dupilumab administration. * Data available for 20 patients.

Figure 1a,b show the changes in FEV_1_ and %FEV_1_ values before and after dupilumab administration. Dupilumab significantly improved FEV_1_ in the 20 patients for whom data were available (median improvement in FEV_1_ of +145 mL; IQR, +88–358 mL; *p* < 0.01). FEV_1_ decreased slightly in 2 patients (−20 mL and −70 mL) but increased in the remaining 18 patients, especially in 7 patients (35.0%) who had an improvement of >200 mL. The %FEV_1_ improved by >5% in 55% of the patients; the median improvement was +5.4% (IQR, +3.0–15.9%; *p* < 0.01). It also significantly improved the ACT scores of 20 patients for whom data were available (Figure 1c). Only one patient had a one-point drop in the ACT score, whereas eight patients had a score improvement of ≥3 points. The median improvement in the ACT score was +2 (IQR, +1–5; *p* < 0.01).

Table 3 shows the efficacy of dupilumab and previous biologics for the treatment of severe asthma based on the GETE. The overall responder rate (excellent/good) following dupilumab treatment was 77.8%. After switching, scores improved in 14 patients (51.9%) but deteriorated in 1 patient. Among the 15 patients for whom an improvement in asthma symptoms was not the reason for the switch to dupilumab, an improved GETE was recorded in 5 patients (33.3%). A decrease in the OCS dose was possible in four of the nine OCS-treated patients.

**Figure 2:**
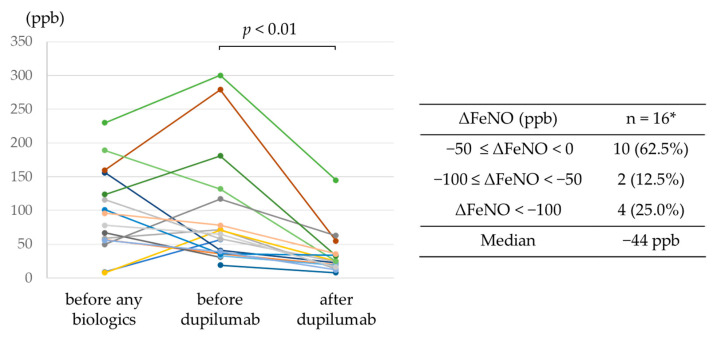
Trends in FeNO levels from before treatment with any biologics to after dupilumab administration. * Data available for 16 patients.

The trends in FeNO levels from before treatment with any biologics to after dupilumab administration are shown in Figure 2. In all 16 patients for whom FeNO levels were available before and after dupilumab administration, dupilumab reduced levels, with a reduction >100 ppb achieved in 4 patients.

Our study included six patients with blood eosinophil counts >1500/μL before treatment with biologics, all of whom were switched from mepolizumab (n = 1) or benralizumab (n = 5). In four, the FEV_1_ increased by >100 mL. In this group, according to the GETE, the overall responder rate (excellent/good) among dupilumab-treated patients was 66.7%.

The GETE-improved and non-improved groups were compared to detect predictors of the efficacy of dupilumab in asthma patients (Table 4). There were no significant differences between the two groups, and no predictors of response based on the GETE in patients switched to dupilumab from other biologics could be identified.

Although our analysis was limited to the 20 patients for whom data were available, the ACT-improved group (+3 ≤ ACT) was compared to the non-improved group (ACT < +3). Both blood eosinophil count before the use of any biologics and FeNO level before the use of dupilumab were significantly higher in the ACT-improved group than in the non-ACT-improved group (Table 5). A comparison of the FEV_1_-improved group (100 mL ≤ ΔFEV_1_) with the non-improved group (ΔFEV_1_ < 100 mL) for the 20 patients for whom data were available did not reveal a significant difference between the two groups (Table 6).

Table 7 shows the efficacy of dupilumab for the treatment of ECRS/CRSwNP as evaluated based on physician assessment. ECRS/CRSwNP improved in 20 of the 23 patients (87.0%) with ECRS/CRSwNP.

### 3.3. Adverse Events

**Figure 3:**
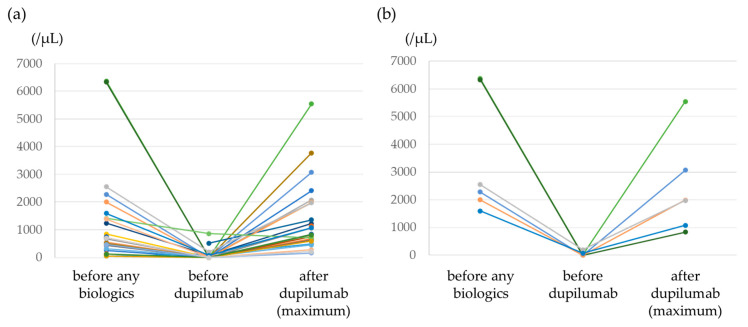
Trends in blood eosinophil counts from before treatment with any biologics to after dupilumab therapy. (**a**) All patients, (**b**) the six patients with blood eosinophil counts >1500/μL.

Figure 3a shows blood eosinophil counts before the use of any biologics, before the use of dupilumab, and after the use of dupilumab. Transient hypereosinophilia >1500/μL occurred in 8 of the 27 study patients (29.6%), and a value >3000/μL was observed in 3 patients (11.1%). The highest blood eosinophil count value was 5546/μL. The median time for the blood eosinophil count to reach its highest value was 182 days (IQR, 113–283 days). All patients with dupilumab-induced hypereosinophilia were asymptomatic. Dupilumab was interrupted due to hypereosinophilia in one patient but was later resumed. One patient had an adverse event of diarrhea, but dupilumab was continued. Figure 3b shows the changes in the eosinophil counts of the six patients with blood eosinophil counts >1500/μL before the use of any biologics. In four of the patients (66.7%), blood eosinophil counts increased to >1500/μL after dupilumab administration. All patients were asymptomatic and were able to continue dupilumab therapy.

## 4. Discussion

A switch to dupilumab therapy has been examined in a few studies. Dupin et al. reported the efficacy of dupilumab in patients with severe asthma, nearly all (97%) with a history of biologics treatment [12]. The study showed significant improvements in ACT scores and FEV_1_ values after dupilumab administration. Another study of 16 patients with a history of biologics treatment found that dupilumab was effective for preventing exacerbations and reducing the steroid dose [13]. However, those studies did not specify the period between discontinuation of the previous biologic and dupilumab administration. Mümmler et al. reported the efficacy of a switch to dupilumab therapy. After the switch, patients had better asthma control, improved lung function, and decreased FeNO, total IgE, OCS dosage, and exacerbation rate [14]. The study allowed up to 6 months between the switch to dupilumab and the discontinuation of a previous biologic.

Despite the demonstrated efficacy of dupilumab in patients with a history of biologics treatment reported in previous studies, it is difficult to compare the efficacy of dupilumab with that of previous biologics due to differences in the interval before dupilumab initiation. The present study, however, was limited to a short switching interval and thus allowed a clear comparison between previous biologics and dupilumab. Our results show that dupilumab improved lung function and symptoms in the absence of a treatment interval following the discontinuation of previous biologics. The switch to dupilumab also led to a reduction in FeNO. In the QUEST study, dupilumab treatment significantly reduced FeNO levels [8], as is also reported for other biologics [23,24,25]. Our results similarly imply that dupilumab is more effective than other biologics in decreasing FeNO levels.

While some patients with severe asthma clearly responded better to dupilumab than to other biologics, we were unable to identify the predictors of a better response. GETE-improved and non-improved groups were compared to identify the predictors of dupilumab efficacy for the treatment of asthma. Blood eosinophil counts and FeNO were promising candidates, as their predictive ability was previously reported [8,9]. Dupilumab resulted in a greater benefit with respect to the prevention of severe asthma exacerbation, an improved FEV_1_, and reduced glucocorticoid use among patients with relatively high blood eosinophil counts and FeNO levels. However, neither blood eosinophil count nor FeNO level could be confirmed as predictive of a response to dupilumab as defined by the GETE. Patients with a high blood eosinophil count or a high FeNO level are more likely to respond to other biologics, as well as to dupilumab [26,27,28,29,30,31]. Therefore, it may be difficult to predict whether dupilumab is more likely to be effective based on these biomarkers. In addition, ECRS/CRSwNP patients have elevated FeNO levels [32]. In the present study, 85.2% of patients had concomitant ECRS/CRSwNP, which may have influenced their FeNO levels, reducing the predictability of efficacy. Nonetheless, a previous study reported that FeNO levels were predictive of efficacy, even in patients who had switched biologics [14]. Our analysis based on the ACT also indicated higher FeNO levels before the use of dupilumab and higher blood eosinophil counts before the use of any biologics in the ACT-improved group than in the non-ACT-improved group. Therefore, while these biomarkers may be candidates, larger studies are needed to identify clear predictors of efficacy in patients who have switched to dupilumab from other biologics.

The efficacy of dupilumab in asthma patients with blood eosinophil counts >1500/μL was not established in the QUEST trial because it excluded these patients at screening [8]. In our study, the six patients with blood eosinophil counts >1500/μL were switched from anti-IL-5/IL-5 receptor antibody to dupilumab, resulting in an improved FEV_1_ and a high response rate (66.7%) based on the GETE. These results imply that dupilumab is a worthwhile treatment option, even in patients with blood eosinophil counts >1500/µL, a condition under which anti-IL-5/IL-5 receptor antibody treatment is preferred.

The high efficacy of dupilumab determined in this study may have been influenced by patient selection. Among our patients, the proportion with ECRS/CRSwNP was very high (85.2%) and was higher than the proportion in severe asthma cohorts enrolled in recent randomized controlled trials (~20%) [8,33]. A post hoc analysis of the QUEST trial data implied higher dupilumab efficacy in patients with complicated chronic sinusitis [33]. Further studies are needed to determine whether dupilumab is equally effective for patients without ECRS/CRSwNP.

We observed that dupilumab improved ECRS/CRSwNP in 20 of the 23 patients (87.0%). Although other biologics (omalizumab, mepolizumab, benralizumab) have also been reported to be effective against ECRS/CRSwNP [34,35,36], the high efficacy of dupilumab in our switched cases implies that it is more effective against ECRS/CRSwNP than the other biologics.

Previous analyses of dupilumab-related adverse events among patients in clinical trials have reported that hypereosinophilia (>3000/μL) developed in 1–13% of them [7,8,37,38]. Most of these events occurred in patients with high baseline eosinophil levels (>500/μL) and were not associated with symptoms or discontinuation of therapy [38]. In our study, 11.1% of patients had eosinophilia >3000/μL, but all were asymptomatic and were able to continue dupilumab therapy. Although hypereosinophilia should be monitored, dupilumab was safe in patients switched from other biologics, even for those with baseline blood eosinophil counts >1500/μL.

Our study had several limitations. First, it was retrospective, was non-randomized, and had a small sample size. Second, some data were missing due to the retrospective nature of the study. Third, because of the high proportion of patients with ECRS/CRSwNP complications, it could not be determined whether our results can be generalized to all patients with severe asthma. Fourth, as 77.8% of patients switched from benralizumab, the efficacy of dupilumab in patients switching from omalizumab or mepolizumab could not be adequately studied. To address these limitations, larger prospective observational studies are needed that stratify patients according to ECRS/CRSwNP status and previous biologics. Despite these limitations, the real-world data provided by our study support the efficacy and safety of a therapeutic switch to dupilumab, particularly for patients with ECRS/CRSwNP.

## 5. Conclusions

Dupilumab is expected to be highly effective for the treatment of severe asthma and ECRS/CRSwNP, even in patients who are switched from other biologics without a treatment interval.

## Figures and Tables

**Table 1:** Clinical characteristics of the 27 study patients.

Characteristics	n = 27
Age, years—median (IQR)	57 (45–68)
Male sex—n (%)	15 (55.6)
Disease duration, years—median (IQR)	12 (8–26)
Body mass index—median (IQR)	24.3 (20.0–27.4)
Smoking history	
Never—n (%)	16 (59.3)
Former or current—n (%)	11 (40.7)
ACT—median (IQR)	20.5 (16–23) *
Allergies—n (%)	16 (59.3)
Comorbidity	
ABPA—n (%)	3 (11.1)
ECRS—n (%)	23 (85.2)
EOM—n (%)	11 (40.7)
AD—n (%)	2 (7.4)
AR—n (%)	10 (37.0)
Treatment	
High dose ICS—n (%)	18 (66.7)
Medium dose ICS—n (%)	9 (33.3)
LABA—n (%)	26 (96.3)
LAMA—n (%)	14 (51.9)
LTRA—n (%)	18 (66.7)
Xanthine—n (%)	8 (29.6)
Maintenance OCS—n (%)	9 (33.3)
Previous biologics	
Omalizumab—n (%)	3 (11.1)
Mepolizumab—n (%)	3 (11.1)
Benralizumab—n (%)	21 (77.8)
Treatment period—median (IQR)	421 (301–673)
Biomarkers (before the use of any biologics)	
Blood eosinophil count (/μL)—median (IQR)	690 (352–1407) *
Serum IgE (IU/mL)—median (IQR)	436 (188–881) †
FeNO (ppb)—median (IQR)	82 (56–116) ‡
FeNO before dupilumab use (ppb)—median (IQR)	60 (37–77) ‡
Pulmonary function	
FEV_1_ (L)—median (IQR)	1.84 (1.50–2.39) ‡
%FEV_1_ (%)—median (IQR)	80.0% (60.1–84.9) ‡
FEV_1_/FVC (%)—median (IQR)	63.5 (54.0–75.3) ‡

* Data available for 26 patients. † Data available for 20 patients. ‡ Data available for 21 patients. Abbreviations: ACT, asthma control test; ABPA, allergic bronchopulmonary aspergillosis; ECRS, eosinophilic chronic rhinosinusitis; EOM, eosinophilic otitis media; AD, atopic dermatitis; AR, allergic rhinitis; ICS, inhaled corticosteroid; LABA, long-acting beta-2 agonist; LAMA, long-acting muscarinic antagonist; LTRA, leukotriene receptor antagonist; OCS, oral corticosteroids; FeNO, fractional exhaled nitric oxide; FEV_1_, forced expiratory volume in one second; FVC, forced vital capacity; IQR, interquartile range.

**Table 2:** Reasons for switching biologics.

Reasons (n = 27)	n (%)
Asthmatic symptoms	5 (18.5)
Asthmatic and ECRS symptoms	7 (25.9)
ECRS symptoms	10 (37.0)
EOM symptoms	3 (11.1)
ECRS and EOM symptoms	1 (3.7)
Self-administration	1 (3.7)

Abbreviations: ECRS, eosinophilic chronic rhinosinusitis; EOM, eosinophilic otitis media.

**Table 3:** GETE score of dupilumab-treated patients (n = 27).

GETE Score	Previous Biologics: n (%)	Dupilumab: n (%)
Excellent	3 (11.1)	6 (22.2)
Good	8 (29.6)	15 (55.6)
Moderate	12 (44.4)	4 (14.8)
Poor	4 (14.8)	1 (3.7)
Worsening	0 (0)	1 (3.7)
Excellent/Good	11 (40.7)	21 (77.8)

Abbreviations: GETE, global evaluations of treatment effectiveness.

**Table 4:** Comparison between the GETE-improved group and the non-GETE-improved group.

	GETE-Improved (n = 14)	Non-GETE-Improved (n = 13)	*p*-Value
Age, years—median (IQR)	62 (52–72)	55 (41–60)	0.14
Male sex—n (%)	10 (71.4)	5 (38.5)	0.13
Body mass index—median (IQR)	24.6 (22.8–27.8)	20.8 (18.5–26.4)	0.26
Never smoker—n (%)	6 (42.9)	10 (76.9)	0.12
ABPA—n (%)	3 (21.4)	0 (0)	0.22
ECRS—n (%)	11 (78.6)	12 (92.3)	0.60
EOM—n (%)	7 (50.0)	4 (30.8)	0.44
Maintenance OCS—n (%)	4 (28.6)	5 (38.5)	0.70
Biomarkers (before the use of any biologics)			
Blood eosinophil count (/μL)—median (IQR)	601 (270–1144)	703 (498–1695)	0.53
Serum IgE (IU/mL)—median (IQR)	395 (296–710)	272 (135–584)	0.43
FeNO (ppb)—median (IQR)	82 (50–124)	87 (57–103)	0.97
FeNO before dupilumab use (ppb)—median (IQR)	71 (49–125)	51 (36–68)	0.39

Abbreviations: GETE, global evaluations of treatment effectiveness; ABPA, allergic bronchopulmonary aspergillosis; ECRS, eosinophilic chronic rhinosinusitis; EOM, eosinophilic otitis media; OCS, oral corticosteroids; FeNO, fractional exhaled nitric oxide; IQR, interquartile range.

**Table 5:** Comparison between the ACT-improved group and the non-improved group.

	ACT-Improved (n = 8)	Non-ACT-Improved (n = 12)	*p*-Value
Age, years—median (IQR)	56 (43–64)	60 (46–73)	0.33
Male sex—n (%)	4 (50.0)	8 (66.7)	0.65
Body mass index—median (IQR)	25.5 (23.0–27.7)	21.2 (19.4–25.0)	0.22
Never smoker—n (%)	5 (62.5)	7 (58.3)	1.00
ABPA—n (%)	2 (25.0)	0 (0)	0.15
ECRS—n (%)	7 (87.5)	9 (75.0)	0.62
EOM—n (%)	3 (37.5)	5 (41.7)	1.00
Maintenance OCS—n (%)	3 (37.5)	2 (16.7)	0.35
Biomarkers (before the use of any biologics)			
Blood eosinophil count (/μL)—median (IQR)	1045 (699–1682)	439 (95–516) *	<0.01
Serum IgE (IU/mL)—median (IQR)	388 (183–710) †	272 (150–433) ‡	0.54
FeNO (ppb)—median (IQR)	87 (40–126)	67 (57–101) §	0.89
FeNO before dupilumab use (ppb)—median (IQR)	68 (58–88)	36 (33–47) ‡	0.04

* Data available for 11 patients. † Data available for 7 patients. ‡ Data available for 8 patients. § Data available for 9 patients. Abbreviations: ACT, asthma control test; ABPA, allergic bronchopulmonary aspergillosis; ECRS, eosinophilic chronic rhinosinusitis; EOM, eosinophilic otitis media; OCS, oral corticosteroids; FeNO, fractional exhaled nitric oxide; IQR, interquartile range.

**Table 6:** Comparison between the FEV_1_-improved group and the non-improved group.

	FEV_1_-Improved (n = 14)	Non-FEV_1_-Improved (n = 6)	*p*-Value
Age, years—median (IQR)	62 (57–69)	46 (44–67)	0.54
Male sex—n (%)	10 (71.4)	3 (50.0)	0.61
Body mass index—median (IQR)	23.2 (20.5–25.8)	27.2 (22.6–32.0)	0.19
Never smoker—n (%)	6 (42.9)	5 (83.3)	0.16
ABPA—n (%)	1 (7.1)	1 (16.7)	0.52
ECRS—n (%)	12 (85.7)	5 (83.3)	1.00
EOM—n (%)	6 (42.9)	2 (33.3)	1.00
Maintenance OCS—n (%)	4 (28.6)	1 (16.7)	1.00
Biomarkers (before the use of any biologics)			
Blood eosinophil count (/μL)—median (IQR)	692 (460–1848)	488 (359–1059)	0.44
Serum IgE (IU/mL)—median (IQR)	395 (157–710) *	188 (150–206) †	0.28
FeNO (ppb)—median (IQR)	73 (55–100) ‡	101 (56–124) §	0.57
FeNO before dupilumab use (ppb)—median (IQR)	68 (57–77) ¶	39 (36–41) §	0.27

* Data available for 11 patients. † Data available for 4 patients. ‡ Data available for 12 patients. § Data available for 5 patients. ¶ Data available for 10 patients. Abbreviations: FEV_1_, forced expiratory volume in one second; ABPA, allergic bronchopulmonary aspergillosis; ECRS, eosinophilic chronic rhinosinusitis; EOM, eosinophilic otitis media; OCS, oral corticosteroids; FeNO, fractional exhaled nitric oxide; IQR, interquartile range.

**Table 7:** Efficacy of dupilumab for ECRS based on assessment of each physician.

ECRS Symptoms (n = 23)	no. (%)
Improved	20 (87.0)
No change	3 (13.0)
Worsening	0 (0)

Abbreviations: ECRS, eosinophilic chronic rhinosinusitis.

## Data Availability

The data presented in this study are available on request from the corresponding author.

## References

[1] Brusselle G.G., Koppelman G.H. (2022). Biologic therapies for severe asthma. N. Engl. J. Med..

[2] Hekking P.W., Wener R.R., Amelink M., Zwinderman A.H., Bouvy M.L., Bel E.H. (2015). The prevalence of severe refractory asthma. J. Allergy Clin. Immunol..

[3] Pelaia G., Gallelli L., Renda T., Romeo P., Busceti M.T., Grembiale R.D., Maselli R., Marsico S.A., Vatrella A. (2011). Update on optimal use of omalizumab in management of asthma. J. Asthma Allergy.

[4] Humbert M., Beasley R., Ayres J., Slavin R., Hébert J., Bousquet J., Beeh K.M., Ramos S., Canonica G.W., Hedgecock S. (2005). Benefits of omalizumab as add-on therapy in patients with severe persistent asthma who are inadequately controlled despite best available therapy (GINA 2002 step 4 treatment): INNOVATE. Allergy.

[5] Farne H.A., Wilson A., Powell C., Bax L., Milan S.J. (2017). Anti-IL5 therapies for asthma. Cochrane Database Syst. Rev..

[6] Gandhi N.A., Pirozzi G., Graham N.M.H. (2017). Commonality of the IL-4/IL-13 pathway in atopic diseases. Expert. Rev. Clin. Immunol..

[7] Maspero J., Adir Y., Al-Ahmad M., Celis-Preciado C.A., Colodenco F.D., Giavina-Bianchi P., Lababidi H., Ledanois O., Mahoub B., Perng D.-W. (2022). Type 2 inflammation in asthma and other airway diseases. ERJ Open Res..

[8] Castro M., Corren J., Pavord I.D., Maspero J., Wenzel S., Rabe K.F., Busse W.W., Ford L., Sher L., Fitzgerald J.M. (2018). Dupilumab Efficacy and Safety in Moderate-to-Severe Uncontrolled Asthma. N. Engl. J. Med..

[9] Rabe K.F., Nair P., Brusselle G., Maspero J.F., Castro M., Sher L., Zhu H., Hamilton J.D., Swanson B.N., Khan A. (2018). Efficacy and safety of dupilumab in glucocorticoid-dependent severe asthma. N. Engl. J. Med..

[10] Wenzel S., Ford L., Pearlman D., Spector S., Sher L., Skobieranda F., Wang L., Kirkesseli S., Rocklin R., Bock B. (2013). Dupilumab in persistent asthma with elevated eosinophil levels. N. Engl. J. Med..

[11] Wenzel S., Castro M., Corren J., Maspero J., Wang L., Zhang B., Pirozzi G., Sutherland E.R., Evans R.R., Joish V.N. (2016). Dupilumab efficacy and safety in adults with uncontrolled persistent asthma despite use of medium-to-high-dose inhaled corticosteroids plus a long-acting beta2 agonist: A randomised double-blind placebo-controlled pivotal phase 2b dose-ranging trial. Lancet.

[12] Dupin C., Belhadi D., Guilleminault L., Gamez A., Berger P., De Blay F., Bonniaud P., Leroyer C., Mahay G., Girodet P.O. (2020). Effectiveness and safety of dupilumab for the treatment of severe asthma in a real-life French multi-centre adult cohort. Clin. Exp. Allergy.

[13] Numata T., Araya J., Miyagawa H., Okuda K., Takekoshi D., Hashimoto M., Minagawa S., Ishikawa T., Hara H., Kuwano K. (2022). Real-World Effectiveness of Dupilumab for Patients with Severe Asthma: A Retrospective Study. J. Asthma Allergy.

[14] Mümmler C., Munker D., Barnikel M., Veit T., Kayser M.Z., Welte T., Behr J., Kneidinger N., Suhling H., Milger K. (2021). Dupilumab improves asthma control and lung function in patients with insufficient outcome during previous antibody therapy. J. Allergy Clin. Immunol. Pract..

[15] Ichinose M., Sugiura H., Nagase H., Yamaguchi M., Inoue H., Sagara H., Tamaoki J., Tohda Y., Munakata M., Yamauchi K. (2017). Japanese guidelines for adult asthma 2017. Allergol. Int..

[16] Global Initiative for Asthma (2019). Asthma Management and Prevention.

[17] Tokunaga T., Sakashita M., Haruna T., Asaka D., Takeno S., Ikeda H., Nakayama T., Seki N., Ito S., Murata J. (2015). Novel scoring system and algorithm for classifying chronic rhinosinusitis: The JESREC Study. Allergy.

[18] Humbert M., Taille C., Mala L., Le Gros V., Just J., Molimard M. (2018). Investigators S: Omalizumab effectiveness in patients with severe allergic asthma according to blood eosinophil count: The STELLAIR study. Eur. Respir. J..

[19] Lloyd A., Turk F., Leighton T., Walter Canonica G. (2007). Psychometric evaluation of Global Evaluation of Treatment Effectiveness: A tool to assess patients with moderate-to-severe allergic asthma. J. Med. Econ..

[20] Nathan R.A., Sorkness C.A., Kosinski M., Schatz M., Li J.T., Marcus P., Murray J.J., Pendergraft T.B. (2004). Development of the asthma control test: A survey for assessing asthma control. J. Allergy Clin. Immunol..

[21] Schatz M., Kosinski M., Yarlas A.S., Hanlon J., Watson M.E., Jhingran P. (2009). The minimally important difference of the Asthma Control Test. J. Allergy Clin. Immunol..

[22] Kanda Y. (2013). Investigation of the freely available easy-to-use software ‘EZR’ for medical statistics. Bone Marrow Transplant..

[23] Kayser M.Z., Drick N., Milger K., Fuge J., Kneidinger N., Korn S., Buhl R., Behr J., Welte T., Suhling H. (2021). Real-world multicenter experience with mepolizumab and benralizumab in the treatment of uncontrolled severe eosinophilic asthma over 12 months. J. Asthma Allergy.

[24] Pelaia C., Crimi C., Benfante A., Caiaffa M.F., Calabrese C., Carpagnano G.E., Ciotta D., D’Amato M., Macchia L., Nolasco S. (2021). Therapeutic effects of benralizumab assessed in patients with severe eosinophilic asthma: Real-life evaluation correlated with allergic and non-allergic phenotype expression. J. Asthma Allergy.

[25] Pianigiani T., Alderighi L., Meocci M., Messina M., Perea B., Luzzi S., Bergantini L., D’Alessandro M., Refini R.M., Bargagli E. (2023). Exploring the interaction between fractional exhaled nitric oxide and biologic treatment in severe asthma: A systematic review. Antioxidants.

[26] FitzGerald J.M., Bleecker E.R., Menzies-Gow A., Zangrilli J.G., Hirsch I., Metcalfe P., Newbold P., Goldman M. (2018). Predictors of enhanced response with benralizumab for patients with severe asthma: Pooled analysis of the SIROCCO and CALIMA studies. Lancet Respir. Med..

[27] Kavanagh J.E., Hearn A.P., Dhariwal J., d’Ancona G., Douiri A., Roxas C., Fernandes M., Green L., Thomson L., Nanzer A.M. (2020). Real-world effectiveness of benralizumab in severe eosinophilic asthma. Chest.

[28] Sandhu Y., Harada N., Sasano H., Harada S., Ueda S., Takeshige T., Tanabe Y., Ishimori A., Matsuno K., Abe S. (2023). Pretreatment frequency of circulating th17 cells and feno levels predicted the real-world response after 1 year of benralizumab treatment in patients with severe asthma. Biomolecules.

[29] Watanabe H., Shirai T., Hirai K., Akamatsu T., Nakayasu H., Tamura K., Masuda T., Takahashi S., Tanaka Y., Kishimoto Y. (2021). Blood eosinophil count and FeNO to predict benralizumab effectiveness in real-life severe asthma patients. J. Asthma.

[30] Menigoz C., Dirou S., Chambellan A., Hassoun D., Moui A., Magnan A., Blanc F.X. (2023). Use of FeNO to predict anti-IL-5 and IL-5R biologics efficacy in a real-world cohort of adults with severe eosinophilic asthma. J. Asthma.

[31] Hanania N.A., Wenzel S., Rosen K., Hsieh H.J., Mosesova S., Choy D.F., Lal P., Arron J.R., Harris J.M., Busse W. (2013). Exploring the effects of omalizumab in allergic asthma: An analysis of biomarkers in the EXTRA study. Am. J. Respir. Crit. Care Med..

[32] Takeno S., Taruya T., Ueda T., Noda N., Hirakawa K. (2013). Increased exhaled nitric oxide and its oxidation metabolism in eosinophilic chronic rhinosinusitis. Auris Nasus Larynx.

[33] Maspero J.F., Katelaris C.H., Busse W.W., Castro M., Corren J., Chipps B.E., Peters A.T., Pavord I.D., Ford L.B., Sher L. (2020). Dupilumab efficacy in uncontrolled, moderate-to-severe asthma with self-reported chronic rhinosinusitis. J. Allergy Clin. Immunol. Pract..

[34] Gevaert P., Omachi T.A., Corren J., Mullol J., Han J., Lee S.E., Kaufman D., Ligueros-Saylan M., Howard M., Zhu R. (2020). Efficacy and safety of omalizumab in nasal polyposis: 2 randomized phase 3 trials. J. Allergy Clin. Immunol..

[35] Han J.K., Bachert C., Fokkens W., Desrosiers M., Wagenmann M., Lee S.E., Smith S.G., Martin N., Mayer B., Yancey S.W. (2021). Mepolizumab for chronic rhinosinusitis with nasal polyps (SYNAPSE): A randomised, double-blind, placebo-controlled, phase 3 trial. Lancet Respir. Med..

[36] Bachert C., Han J.K., Desrosiers M.Y., Gevaert P., Heffler E., Hopkins C., Tversky J.R., Barker P., Cohen D., Emson C. (2022). Efficacy and safety of benralizumab in chronic rhinosinusitis with nasal polyps: A randomized, placebo-controlled trial. J. Allergy Clin. Immunol..

[37] Bachert C., Han J.K., Desrosiers M., Hellings P.W., Amin N., Lee S.E., Mullol J., Greos L.S., Bosso J.V., Laidlaw T.M. (2019). Efficacy and safety of dupilumab in patients with severe chronic rhinosinusitis with nasal polyps (LIBERTY NP SINUS-24 and LIBERTY NP SINUS-52): Results from two multicentre, randomised, double-blind, placebo-controlled, parallel-group phase 3 trials. Lancet.

[38] Wechsler M.E., Klion A.D., Paggiaro P., Nair P., Staumont-Salle D., Radwan A., Johnson R.R., Kapoor U., Khokhar F.A., Daizadeh N. (2022). Effect of dupilumab on blood eosinophil counts in patients with asthma, chronic rhinosinusitis with nasal polyps, atopic dermatitis, or eosinophilic esophagitis. J. Allergy Clin. Immunol. Pract..

